# Methodology for the knowledge-based selection of occupational exoskeletons

**DOI:** 10.1007/s11740-025-01338-x

**Published:** 2025-03-20

**Authors:** Tobias Drees, Lennart Ralfs, Benjamin Reimeir, Kai Lemmerz, Robert Weidner, Bernd Kuhlenkötter

**Affiliations:** 1 Chair of Production Systems (LPS), Ruhr-University Bochum, Industriestraße 38c, 44894 Bochum, Germany; 2 Chair of Production Technology, Institute of Mechatronics, University of Innsbruck, Technikerstraße 13, 6020 Innsbruck, Austria; 3 RIF Institut für Forschung und Transfer e.V., Joseph-von-Fraunhofer Straße 20, 44227 Dortmund, Germany; 4 Chair for Automated and Autonomous Systems (AAS), TU Bergakademie Freiberg, Akademiestraße 6, 09599 Freiberg, Germany; 5 Laboratory of Manufacturing Technology, Helmut Schmidt University, Holstenhofweg 85, 22043 Hamburg, Germany

**Keywords:** Occupational exoskeletons, Knowledge-based selection, Methodology design, Multicriterial evaluation, Co-simulation model, Human–robot-collaboration

## Abstract

Occupational exoskeletons for industrial workplaces hold significant promise for improving worker ergonomics and safety. However, the successful selection of an exoskeleton depends on informed decision-making processes that consider various factors ranging from biomechanical performance to usability and compatibility with work tasks. This paper presents a methodology that aims to develop a co-simulation-based selection tool for selecting an exoskeleton for specific industrial work tasks. It integrates multidisciplinary knowledge from biomechanics, human factors engineering, and industrial ergonomics for assessing the suitability of exoskeletons across diverse industrial applications. The methodology is designed as a stage-gate process with five main stages corresponding to the product development process. It describes the main tasks in each phase, their results, and the gates between the stages. The tasks and results are derived and detailed from the current literature and preliminary work. The gates include the specification of the simulation and decision-relevant input and output parameters, the design of the co-simulation model consisting of task and biomechanical simulation, the weighting of the individual decision criteria, and the subsequent implementation of the multi-criteria decision analysis to create a ranking of suitable exoskeletons. This work concludes by elaborating on the impact of the novel co-simulation methodology on research and industry. Research implications include advanced simulation methods for exoskeleton evaluation, the systematic comparison of different exoskeletons, and the development of decision analysis models. Benefits to the industry include improved compatibility, informed selection processes, reduced investment risks, and increased technology adoption.

## Introduction

The interaction of humans and technology in industrial workplaces has intensified in recent years. Exemplary approaches are systems based on human–robot collaboration or exoskeletons supporting the user’s musculoskeletal system [1]. Occupational exoskeletons (OE) are of interest to companies aiming to reduce the strain on workers by supporting the enabling, empowering, facilitating, or adding of movements and stabilizing their posture, thereby providing relief [2]. This interest stems from the goal of preventing long-term strain-related injury to workers, prompting the exploration and adoption of new approaches to support the workforce [3]. However, the adoption rate of OE in industrial workplaces remains low [4], and selecting a suitable OE for the defined task depends on various interdependent constraints that impede the selection process itself [5]. Most crucially, the OE may not be compatible with the user or the specific tasks, tools, or work environment of the intended use case, rendering it ineffective or impractical for use in real-world conditions, which is amplified by the fact that selection and tests often run under laboratory conditions and pursue heterogenous evaluation focusses and methods [4].

Currently, in the industry the selection process predominantly involves presentations by sales representatives, wherein emphasis is placed on ergonomic benefits rather than on the limitations of the OE. Thus, recommendations for selecting suitable OEs are mostly subjective and influenced by affiliations and, therefore, require critical consideration. Furthermore, there is often only limited opportunity for comprehensive testing of different systems during the selection process of OE. While there may be an initial testing period, the variety of OE tested is limited. This adds to the issues of inconsistent data or information on user acceptance, task suitability, and effectiveness of OEs [6].

**Fig. 1:**
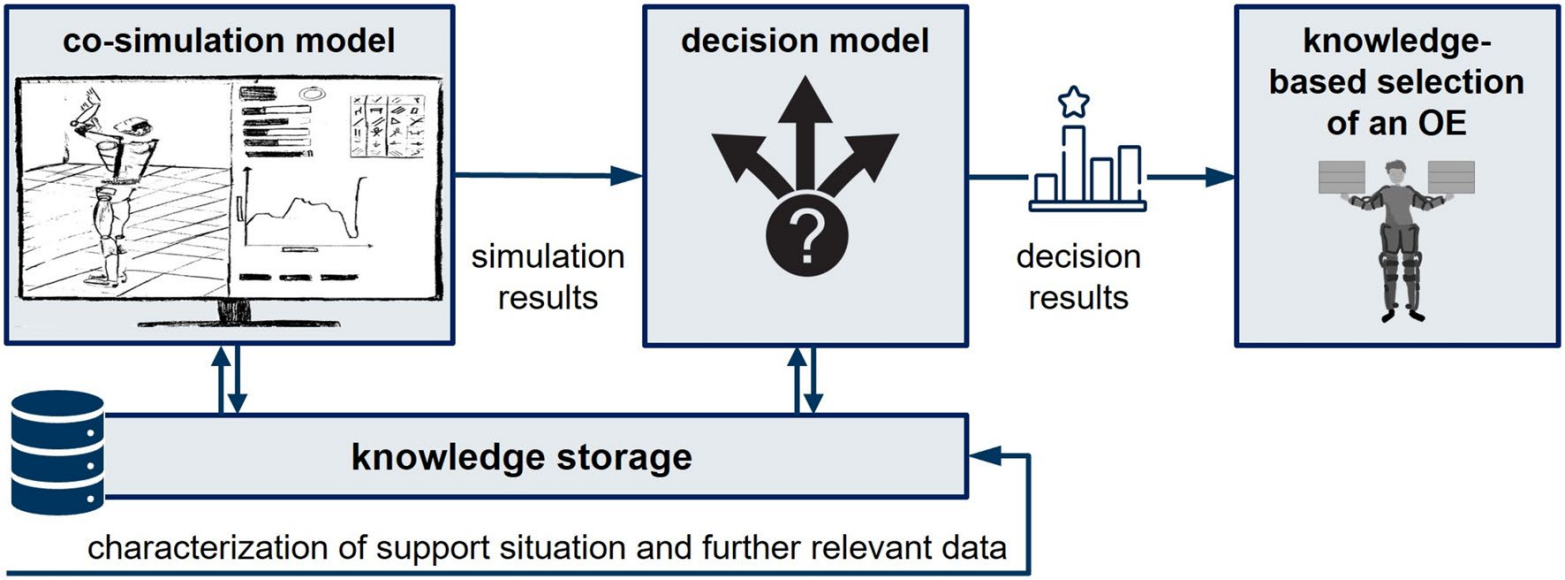
Overview of the desired link between the different simulations and input factors

Despite many unsuccessful implementations [7], the utilization of simulation-based or knowledge-based selection methods remains limited. Thus, there is a need for a more systematic approach that leverages simulation and knowledge-based techniques to address the different aspects and multicriteria decision-making. Consequently, a simulation-based approach that integrates user anthropometrics, task characteristics, and exoskeleton features within a co-simulation model, which is defined as a combination of two or more different simulation models with a different focus, offers numerous advantages for OE selection. This method must facilitate adaptation to specific users and job requirements while enabling comprehensive evaluation of ergonomics, biomechanics, and performance metrics. Moreover, it must enhance efficiency by optimizing decision-making and reduce costs. Figure 1 gives an overview of the desired link between the different simulations and input factors.

Thus, the aim of this paper is to derive and describe a methodology for creating a simulation-based tool for selecting suitable OE, considering various heterogeneous economic, ergonomic, and biomechanical criteria valid for diverse use cases. The methodology results in creating a selection tool for the industry. The following research questions arise:

- What is the current status quo on selecting a specific OE for an industrial use case?
- What stages and tasks are necessary to develop a knowledge-based OE selection assistance tool?
- What are the expected benefits for research and industry resulting from a knowledge-based OE selection?

The remainder of this work is structured as follows. Section 2 deals with the state of the art regarding OE selection, the simulation of the effects of their use, and further simulation input factors. Section 3 merges the findings to create a list of requirements for the methodology, while Section 4 conceptualizes the OE selection tool. Sect. 5 creates the methodology for its development. It further structures the development process and describes specific tasks and (intermediary) results. Section 6 derives implications for research and industry and Sect. 7 finishes the work with a conclusion and outlook.

## State of the art

### Methods for the selection of OEs

Various methods for selecting a suitable OE range from objective and scientific approaches utilizing databases and weighting factors to more subjective evaluations based on checklists and visualizations (see e.g., [8–12]). The methods and approaches either pursue the goal of user-centered human-technology interaction or evaluate and select suitable OEs for support situations. However, their core focus is providing generic recommendations for using OEs, rather than a knowledge-based individualized selection. Because of the diversity of criteria and categories used, clear guidance for decision-makers remains elusive, highlighting the need for further research and development in this area [5].

An approach for the selection of an OE in an industrial environment is presented in [13]. Thereby, *ExoMatch* is an objective method considering, filtering, and matching significant attributes from OE to workplaces to select the most suitable technical system. A comparable approach is the decision support matrix by Ralfs et al. which promotes a suitability assessment through combined consideration and evaluation of matching task characteristics and technical properties of OEs [9]. Further studies also provide additional decision-making aids and recommendations with regard to transfer into practical application [14]. Another decision tool enables the integration of OEs into existing production systems based on virtual reality techniques [15]. This approach comprises the validation and finetuning as well as the decision support for evaluating the fitness of prototypes and the effective and safe operator training within the application. However, no evaluation approach regarding ergonomics has been integrated in this case. Constantinescu et al. present a methodology for the simulation of OEs in Siemens Process Simulate as digital twins [16]. The approach is based on the geometric extension of the human model *Jack* by elements of a passive exoskeleton, carrying out an ergonomic evaluation of manual work activities with and without physical support. However, only the anthropometric behavior and fewer methodologies for biomechanical evaluation and independent selection are considered [17]. Golabchi et al. propose a framework that assists organizations in successfully evaluating and adopting OEs [18]. They cite Crea et al. [4] regarding that the large-scale deployment of OE requires a stepwise knowledge-based approach. However, the selection stage remains vague. It is based on selecting the body parts that should be assisted as well as the OE type and provides some key characteristics to be evaluated. How the selection is performed and how differences and interdependencies are dealt with in the decision model are not covered [19]. Mark et al. describe a methodology to find appropriate worker assistance systems. Their focus lies on ranking different assistance systems (e.g., eye tracking, arm exoskeleton, portable computer, among others) with the help of 23 parameters to describe tasks, user groups, and the support of the systems, but no differentiation between OEs is made [20].

Moreover, a major barrier to selecting an OE is the lack of research investigating their effectiveness and usability in real workplaces [18]. Numerous laboratory-based studies show only limited relevance to industrial context. However, in recent years, the number of field studies has increased as the number of different OEs reaching technological maturity has also increased [6]. Nevertheless, field studies are constrained by small sample sizes and emphasize predominantly subjective metrics [4]. Furthermore, a specific OE is already selected when testing starts, hence neglecting the selection process itself [18]. Even if different OEs are compared within a field study (see, e.g., [21]), the final selection relies on the time-consuming, costly, and mostly subjective testing phase, aggravating the decision-making process.

In summary, while some methods offer promising avenues for OE selection, challenges persist, particularly in integrating ergonomic evaluations and addressing the diversity of selection criteria. In addition, the scarcity of research on OE effectiveness and usability in real workplaces underscores the need for further investigation to facilitate informed decision-making in OE selection processes. However, in the various methods and implementation plans, ergonomic and biomechanic evaluations, as well as considerations of task and workplace properties, prove to be key influencing factors for the selection process alongside the characteristics of the OE. Thus, the following sections describe the current state of research in these areas in relation to the selection of OE.

### Characterization of support situation

A support situation for the use of OEs can be characterized by the triad of “activity”, “technology”, and “human”. This triad emphasizes the necessity to align the specific tasks and motions involved in the activity with the capabilities of the exoskeleton technology and the physiological and ergonomic needs of the human user. Achieving this alignment ensures that the OE provides effective support. Hence, the following sections describe the state of the art in the context of OEs for each field mentioned above [22].

#### Human characteristics: ergonomic and biomechanical evaluation based on simulation models

In interaction with digital human models, appropriate methods of virtual ergonomics enable the preventive occupational safety of employees [23]. This allows to evaluate hazardous and physically stressful activities in the early planning process [24]. Various tools, e.g., the *ema Work Designer (emaWD - imk automotive)*, *DELMIA (Dassault Systems)*, or *Process Simulate(Siemens)*, exist for planning manual or hybrid production systems in which digital human models are integrated. Well-known examples of digital human models in the field of ergonomics are *Human Builder* [25], *RAMSIS* [26], *Jack* [27], and *ema* [28].

Furthermore, various commercial or open-source tools and models are available to evaluate biomechanical effects in the context of human motions. The *AnyBody* technology comprises musculoskeletal models and associated software to simulate the human body concerning its working environment [29]. It is used in product design, especially in the automotive, medical, and aerospace industries. Another common framework for the design of musculoskeletal models is the open-source software *OpenSim* [30]. It has been applied to model various anatomical structures from comprehensive full body, lower, and upper limb models to more detailed models of specific structures (e.g., knee, hand, mandibular joint) and can be used to generate accurate simulations of the gait [31]. In the *MATLAB* environment, the *Biomechanics of Bodies (BoB)* tool provides a package of biomechanically relevant functions suitable for performing inverse dynamics analysis and optimization methods to solve muscle force distributions and produce user-friendly graphical image and video output [32] Various comparisons of the software tools reveal differences in the underlying musculoskeletal models and muscle parameters (Hill-type muscle models) based on the anatomical studies it was constructed on [33]. Additionally, variations in computational procedures lead to deviating results in the biomechanical analysis of movements [34]. Better investigation and understanding of the underlying optimization solvers, which are not fully published for all software tools, could lead to more consistent simulation outputs among the different software tools [34]. Biomechanical simulations of human–exoskeleton interaction showed medium to high validity for upper and lower body exoskeletons using either *AnyBody* or *OpenSim* as simulation tools [35]. Depending on the requirements of the simulation, the different tools show various levels of testing against observational data, hence making certain simulation tools more reliable for a specific use case. In addition, the literature contains approaches to co-simulation that focus on human-technology interaction and expand the ergonomics and biomechanics-oriented perspective to include activity-oriented aspects. Existing model-based approaches focus, e.g., on the interaction between humans and OEs [36], and with tools used [37], or the technical design and control of systems [38].

A recent research project, *DigitalExonomics*, integrates digital work process modeling with musculoskeletal simulations to assess OEs. The project employs digital human modeling, motion capture, and biomechanical simulations using *ema* and *AnyBody* to analyze physical strain and evaluate the effectiveness of OEs in industrial applications. The approach enables a personalized and data-driven ergonomic assessment, incorporating both task-specific digital work planning and musculoskeletal load analysis [39].

#### Task and workplace properties: evaluation of work activities

The different support situations require a variety of OEs to assist users [40]. Accordingly, OEs have different morphologies and functional properties, corresponding to functional principles. They are divided according to their supported tasks (e.g., overhead work, prolonged standing, carrying (handling) heavy or bulky objects, or walking (gait assistance)) and, depending on their design, they support different body parts [41], e.g., arms, legs, or the full body [42].

As OEs support the users in varying tasks and with different support characteristics, existing models for manual work activities and automation processes help describe possible use cases. However, these task descriptions only allow a rough allocation to the OE type while neglecting the specific motions, movement durations, and working environments unique for each task. For a more detailed description, manual work activities can be described in various ways as briefly outlined in the following section [43]. Process descriptions or flowcharts can describe the task with sequencing process elements. Nowadays, especially two techniques are well-established: the *work factor method* [44] and the modular systems of the *methods time measurement (MTM)* approach [45]. Moreover, the pen-and-paper-based observational methods in assessing ergonomic risk factors (e.g., *Ovako Working Analysis System OWAS* [46], *Rapid Upper Limb Assessment RULA* [47], or *European Assembly Worksheet EAWS* [48]) further distinguish different motions and identify the ones with the highest ergonomic risk factor [49]. These methods assess postures to evaluate risk factors for work-related musculoskeletal disorders, while the observations are inexpensive and easy to use at different workplaces [50]. However, the methods are based on subjective observation and are prone to error, as nearly 30% of the assessments are erroneous, with some of those errors being severe and completely invalidating the evaluation results [51]. Furthermore, a single method does not cover all risk factors, including working posture, tools, forces, frequency of action, duration, and working environment [49], and a sequence of movements cannot be mapped or evaluated.

Furthermore, Baltrusch et al. [52] provide a list of twelve different realistic working tasks (e.g., lower lifting and forward bending, among others) with a description of their specific characteristics and possible outcome measures that can be compared. In further work, the reliability of the approach for functional evaluation of the OE was assessed [53]. Though promising results, the need for alternative testing approaches (biomechanical and physiological testing, e.g., monitoring metabolic cost) is highlighted [53].

For capturing movements and subsequently evaluating the work activities, motion capture systems are used (e.g., [54]). Different systems are available, mostly based on cameras [55], or inertial trackers [56]. Their common goal is to accurately capture and analyze human movement data in real-time or for later analysis. These systems provide detailed insights into the biomechanics and kinematics of workers, including joint angles, forces exerted, and muscle activations.

Different approaches use or adapt the methods for the use with OEs. Examples are the software *Scalefit* which illustrates the measurable relief when using a shoulder OE based on motion capture [57], the *exoIQ app*, which provides live evaluation of posture according to existing ergonomics catalogs when using the exoskeleton [58], or an adaption of the EAWS by *Fondazione Ergo-MTM Italia* that studies how the ergonomic risk assessment index changes with the use of a passive exoskeleton supporting shoulder movements [59]. They developed an adopted worksheet with lower risk scores for using two different exoskeletons (*Comau MATE* and *Paexo Shoulder*) [59]. The existing software mentioned above can transform the captured data into a risk analysis based on the pen-and-paper methods [28]. Additional methods for evaluating work activities have been developed, including the *ExoLiFFT* method by Zelik et al. [60] and the *ExoWorkathlon* test format [61]. The *ExoLiFFT* method presents a practical and efficient low-barrier approach for assessing the effectiveness of back-support exoskeletons [60]. In contrast, the *ExoWorkathlon* test format provides a standardized setup for evaluating OEs in industry-related and realistic work scenarios [61]. These approaches contribute to the objective assessment of OEs by offering structured, reproducible, and application-oriented evaluation protocols.

#### Features of occupational exoskeletons

Information on, e.g., torque curves and range of motion including angular range is required to specify exoskeletal features and behavior and implement these in a selection tool. In this respect, OEs differ in their force paths (e.g., force transmission from the upper arm to the pelvis [62], from the tool into the upper body [63], or the tool to the ground [64]), translatory or rotatory degrees of freedom, and user interface designs [65]. Additionally, the systems consist of different materials, sensors, and actuators and, thus, apply different actuation principles [66]. These differences pose complex requirements for testing the support provided by OEs and impede standardized test procedures that make a comparison possible between different systems [19]. However, Watterworth et al. describe a test environment to quantify the support provided by four passive upper limb OEs across their range of motion and estimate the degree of support as a function of shoulder elevation angle [67]. Within their quasi-static measurements, they exclude occurring hysteresis in a dynamic setting. Further tests for assessing the torque are conducted by Madinei et al. [68], Hartmann et al. [69], Ito et al. [70], and Klankers et al. [71]. Tröster et al. propose a generic approach that categorizes OE support into four distinct modes based on varying torque characteristics [72]. This structured approach enables a more precise and comparative evaluation of the effects of different OEs, facilitating a detailed analysis of their impact on user performance and ergonomics. Thus, such torque curves present a promising way of comparing OEs with similar support mechanisms but must still be added by other factors such as velocity of movements or range of motion.

Even though the features of OEs might be great for a specific workplace, a high acceptance among the users is necessary to increase the adoption rate of the systems in industry [73]. Recent advancements in OE technology aim to enhance personalized support by incorporating adjustable force profiles, adaptive actuation, and real-time biomechanical feedback. While some systems allow manual adjustments based on user preferences and thus improve acceptance, fully adaptive OEs remain limited due to technological constraints and complexity. According to the technology acceptance model, the acceptance is strongly influenced by the users’ feelings about whether the selected OE actually assists or rather hinders performing the tasks (perceived usefulness), and whether the use feels natural (perceived ease of use) [74]. These soft factors and the resulting usability are difficult to analyze or simulate [75] but important for the selection process [5]. The design of the physical human–machine interfaces (e.g., positions of padding, padding material, softness, number of contact points) affects usability [76] and resulting interaction forces might cause discomfort at key contact points. While increasing support improves biomechanical relief, excessive force transmission may lead to pressure discomfort and movement restrictions, reducing long-term usability. An optimal balance between support and acceptable discomfort is critical for ensuring sustained user compliance and effective OE implementation in industrial settings [77]. Efforts are made to establish consensus regarding the definitions of usability attributes, alongside proposing methods for analyzing usability across various tasks [78]. Surveys on usability attributes capture usability scores for different OEs [79]. However, comparing different systems remains difficult since the survey questions, performed tasks, user groups, and settings vary [80]. Nevertheless, the acceptance and usability resulting from OE features and user perceptions are important factors for decision-making [81].

### Interim conclusion on the state of the art

To sum up, various approaches allow the modeling of tasks and human characteristics in the context of ergonomics improvement and OE introduction. However, comparing the individual results after applying the methods in different settings remains difficult. Pen-and-paper-based task descriptions only allow a rough allocation to the OE type while neglecting the specific motions, movement durations, and working environments unique for each task. Motion capture is accurate and reliable, while allowing the assessment of biomechanical effects when linked to a human model but does not in itself provide results regarding the quality of fit of an OE. In this respect, existing simulation approaches neither allow interactively modeling and ergonomically evaluating OEs to the required extent nor checking accessibility and possibilities of movement executions. Until now, OE evaluation has only been based on prior physical implementations. Thus, positive and negative results can only be obtained after a time- and money-consuming implementation (ex-post evaluation). However, the various methods, results, and data presented in the sections above provide an important basis for the development of a novel simulation-based OE selection tool and form a knowledge storage that requires exploitation. Thus, this work is based on, further develops, and synthesizes state-of-the-art methods to assess the suitability of OEs for given support scenarios before their use.

## Requirements for the methodology

**Table 1:** Requirements for the methodology

Requirements	
Comprehensive data integration	Allow integration of data from various sources, including scientific literature, technical specifications, and manufacturer information
	Incorporate biomechanical and task-specific analysis techniques to assess the impact of OEs
	Include methods for assessing the usability of exoskeletons in real-world industrial environments
	Integrate economic and HMI factors
Decision support	Consider methods from multi-criteria decision analysis (MCDA) for evaluating different OEs
Standardization	Implement the design of standardized data transfer, evaluation protocols, and procedures for assessing the relevant input and output factors
Continous improvement	Allow iterative refinement based on feedback and technological advancements, ensuring its relevance and effectiveness over time
Scalability	Accommodate different types of OEs and allow adaptation to various industrial contexts, allowing for broad applicability and practical implementation across different industries and organizations

Addressing the challenges within the selection process requires collaboration between manufacturers, researchers, occupational health professionals, and users to ensure that OEs satisfy the needs of workers while maximizing safety, productivity, and comfort. A knowledge-based selection tool integrating findings of these different domains promises to better understand the interdependencies and, thus, improve the quality of decision-making. As becomes obvious, the selection of an OE requires a complex and multi-stage consideration of various heterogeneous dimensions, which are relevant at different times in the selection process and have an overall impact on the suitability and effects of an OE. Hence, the methodology for creating the knowledge-based planning tool must fulfill different requirements.

First, comprehensive data integration is essential for creating a robust and reliable system. This includes the ability to integrate data from various sources, such as scientific literature, technical specifications, motion data, and manufacturer information. It should also incorporate biomechanical and task-specific analysis techniques to accurately assess the impact of OEs in different tasks and working conditions. Moreover, the methodology must allow the integration of usability evaluation in real-world industrial environments and other soft as well as economic factors for a holistic assessment. For decision support, the methodology should utilize methods from multi-criteria decision analysis (MCDA). This approach helps evaluate different OEs based on various criteria, enabling a balanced and informed decision-making process that considers multiple perspectives and factors, such as performance, cost, and user satisfaction. Furthermore, standardization is a key requirement. Standardized data storage, access and transfer protocols ensure consistency and reliability in assessing relevant input and output factors. This standardization will facilitate comparisons across different studies and applications, making the system more versatile and trustworthy. In addition, continuous improvement is vital to maintain the system’s relevance and effectiveness over time. This involves allowing for iterative refinement based on user feedback and technological advancements, ensuring that the system evolves in response to new insights and OE developments. In that aspect, scalability is necessary to accommodate different OE types and adapt to various industrial contexts. The methodology must consider this adaptability to ensure that the planning tool can support diverse needs and requirements, enhancing its utility and impact in improving workplace ergonomics and productivity. For the methodology, the following requirements can be derived (cf. Table 1).

## Structural design of the selection tool, its key elements and benefits

As stated above, the support situation for the use of OEs can be characterized by the triad of activity, technology and human. This triad is in line with the dimensions task and workplace properties, user characteristics and exoskeletal features of OE selection criteria published in previous work [5]. Hence, for a knowledge-based selection approach, these dimensions, are in the spotlight due to their direct relevance to the design, functionality, and performance of the OE. For the task and workplace properties, various factors such as task dynamics, load weight, frequency, range of movements, tool usage, and environmental conditions must be considered. Moreover, a knowledge-based approach should allow the consideration of human characteristics, such as anthropometrics, percentiles, movement patterns, postures and individual performance capabilities. It should further grant the evaluation of biomechanical factors, such as joint angles, muscle forces, and load distribution. Understanding the user characteristics is the basis for simulating the interaction of an OE and the user. It analyzes how well the OE fits and supports the user’s body as well as the potential risks of musculoskeletal stress or discomfort during prolonged use. The exoskeletal features include evaluating factors such as the provided torque by the exoskeleton, its mechanical structure, weight, size, power source, actuation principles, and sensor technology. By simulating these aspects, decision-makers gain insights into how different exoskeleton designs and functionalities interact with the specific user and the work environment [9].

While these three dimensions are the primary focus in a simulation-based approach, it is important to recognize that the other criteria dimensions also play significant roles in the decision-making process. However, they may not be as directly amenable to a simulation-based analysis. Nevertheless, soft factors such as user preferences, acceptance, and usability, which require empirical testing and user feedback that can be stored in the knowledge storage, must be included in a decision model as it influences how users think about and interact with the OE. Andrade and Nathan-Roberts [82] summarized key measures for acceptance and adoption within their review that can be implemented in a decision model. The economic dimension influences the financial feasibility of adopting OEs within the organization. However, the economic dimension has no direct impact on the specific fit of an OE to the user and the task where it supports. Moreover, economic considerations involve multiple variables and factors beyond the scope of a typical simulation model. Factors such as market dynamics, labor costs, and organizational budget constraints are highly dynamic and context-dependent, making it challenging to accurately model or simulate [83]. Thus, the economic dimension should be implemented in a decision model but must not be part of a simulation model.

Other dimensions of selecting factors [5] are considered out of scope for a knowledge-based simulation approach. This includes socio-cultural factors (e.g., cultural attitudes, social norms, and perceptions surrounding the use of OEs in the workplace) and political factors encompassing organizational policies, regulations, and stakeholder interests. While political factors and attitudes towards OEs influence the acceptance and adoption of OEs in general, they have no influence on the specific fit of an OE to the task and user involved.

**Fig. 2:**
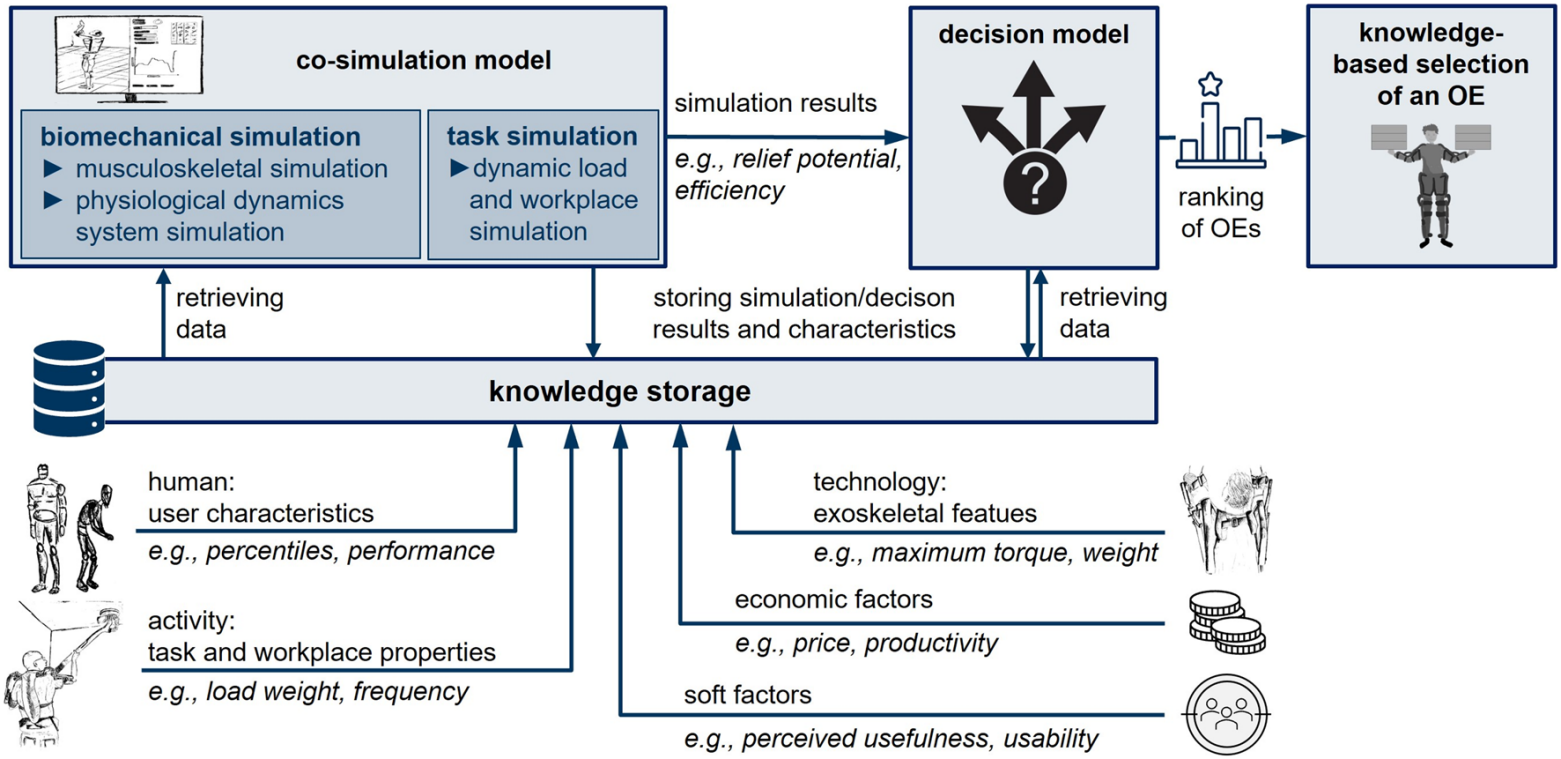
Overall concept of simulation-based OE selection

Figure 2 illustrates the structure of the envisioned knowledge-based selection tool, which is established based on the insights derived so far. It visualizes its main elements, i.e. knowledge storage, co-simulation model, decision model, input parameters and results, and the relationship between them. The co-simulation model that puts a focus on task simulation and biomechanical simulation retrieves the data of the main input factors from the knowledge storage in a standardized way. The biomechanical simulation can be further subdivided into a musculoskeletal simulation that simulates the performed work tasks with an integrated musculoskeletal - exoskeleton model and a physiological dynamics system simulation that simulates the effects on, e.g., energy metabolism and fatigue over time using the musculoskeletal simulation outputs. The task simulation recreates the specific task performed in a support situation from a wide range of tasks performed in industrial environments, especially when no motion capture data is available.

As a result of the simulation layer, intermediate output parameters describe the respective support situation with factors as, e.g., muscle activities, relief potential, efficiency, metabolic demands, movement restriction or assisted torque curve. These results are passed on to the decision model, which further incorporates the economic and soft factors as input parameters. The decision model results in a multifaceted value ($\hbox {Exo}_{value}$) that shows a suitability ranking for OEs based on decision-relevant parameters in the decision model. In its easiest form, $\hbox {Exo}_{value}$ is formed by the product sum of the individual criteria weights and their respective value. It serves as the main output for the tool.

A key challenge remains in outlining the methodology for creating the knowledge-based selection tool depicted in Fig. 2 systematically and establishing effective connections among its diverse input dimensions. Therefore, it is imperative to systematically delineate the stages involved in addressing this challenge. For this purpose, the adoption of a stage-gate process provides a structured framework to ensure that each stage of the methodology is carefully defined, assessed, and validated before progressing to subsequent stages [84]. Thus, the following sections explain the individual stages of the methodology, outline the specific tasks and activities included in each stage, and discuss the criteria for advancing through the quality gates. These efforts aim to ensure the successful development and implementation of the selection tool tailored for OE selection.

**Fig. 3:**
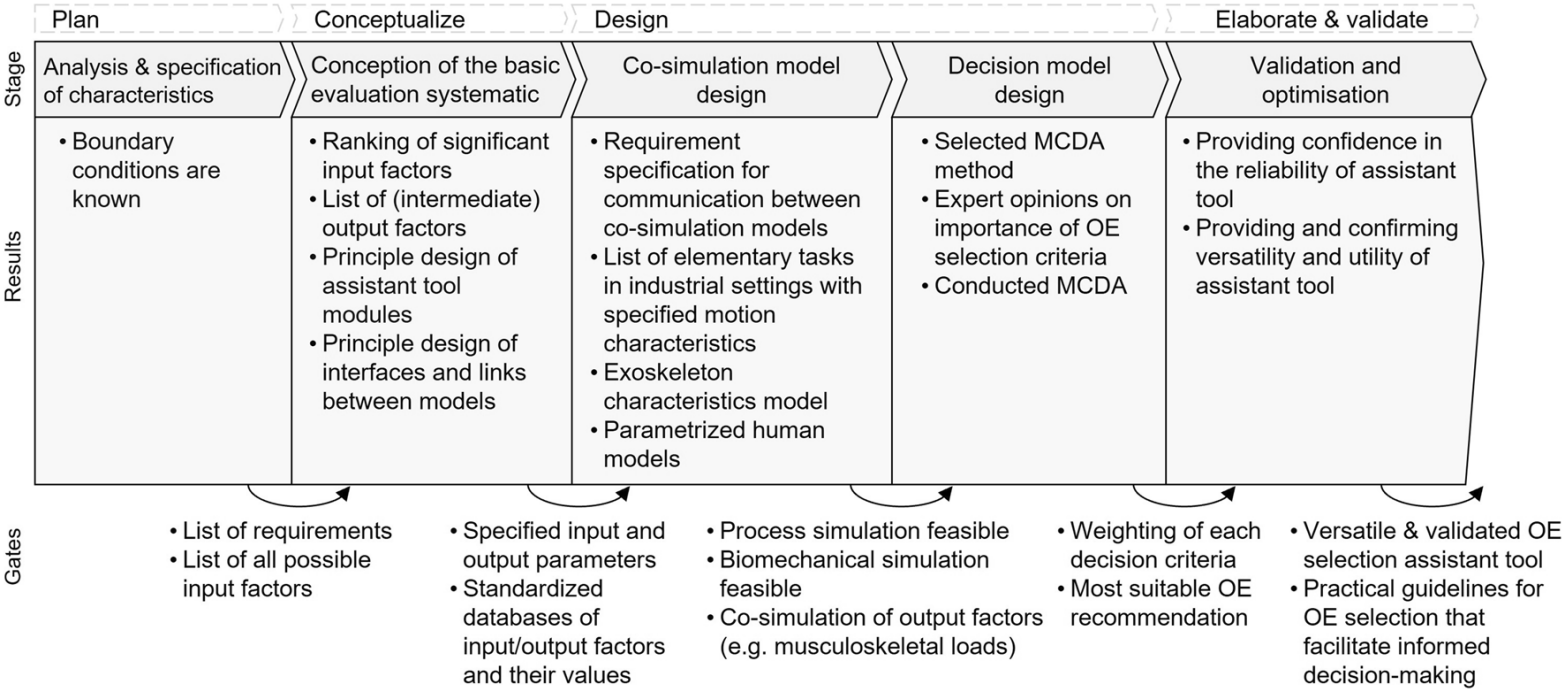
Proposed stage-gate process

## Deriving the methodology

Figure 3 shows an overview of the proposed stage-gate process with its stages, the main results of each stage and the quality gates between the stages. The main phases of the product development process, i.e., plan, conceptualize, design, elaborate, and validate (cf. [84]), provide a systematic structure for the development of the selection tool. Five main stages are derived, which are detailed below:

- analysis and specification of characteristics,
- conception of the basic evaluation systematic,
- co-simulation model design,
- decision model design, as well as
- validation and optimization.

### Plan: analysis and specification of characteristics

**Fig. 4:**
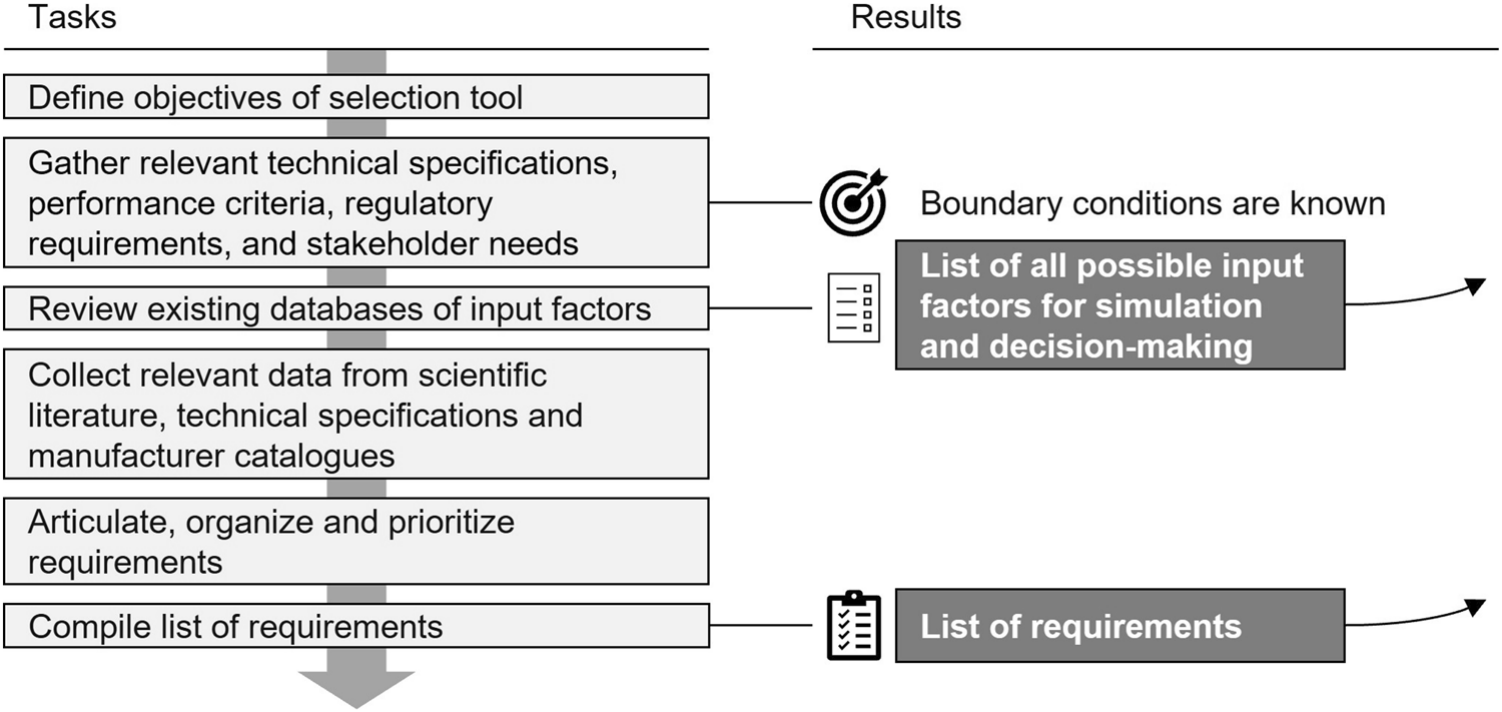
Tasks and results of stage *Analysis and specification of characteristics*

In the initial stage *Plan*, several successive tasks must be performed to analyze and define the scope of the OE selection tool and provide a profound understanding of the support situation. It concludes by specifying the requirements for the tool. Figure 4 gives an overview of the *Plan* stage.

First, the overall objectives must be defined to provide a clear direction for the subsequent steps. Relevant technical specifications, performance criteria, legal requirements, industry standards, and stakeholder needs are comprehensively recorded based on relevant literature. This helps gain insights into constraints crucial for the design process and identify potential challenges or opportunities.

Next, existing databases of input factors are analyzed and matched to the dimensions shown in the overall concept in Fig. 2. This results in a list of all possible input factors. For these, relevant data on OEs must be acquired, which is facilitated through scientific reviews and publications, technical specifications, manufacturer information (such as data sheets), or catalogs like the *Exoskeleton Report* website [41]. Relevant preliminary work can be built on, in which already usable fundamentals are gathered, such as databases and collections of characteristics for the specification of user characteristics, for the description of activity profiles and task properties, and commercial exoskeletons and their characteristic (e.g., [85]).

Once a comprehensive data set, which functions as a knowledge storage, is gathered, the next step is to formulate, organize, and prioritize the requirements. For a simulation-based OE selection tool, the core requirements will arise from the industrial task, workplace properties (e.g., typical handling loads, postures, movement patterns), the user characteristics (e.g., anthropometrics), and the considered OE (e.g., path of force retransmission, torque curve characteristics). The culmination of this stage results in the detailed specification of the characteristics of the selection tool in the form of a requirement list. This comprehensive compilation serves as a basis for the subsequent stages of tool development.

### Conceptualize: conception of the basic evaluation systematic

**Fig. 5:**
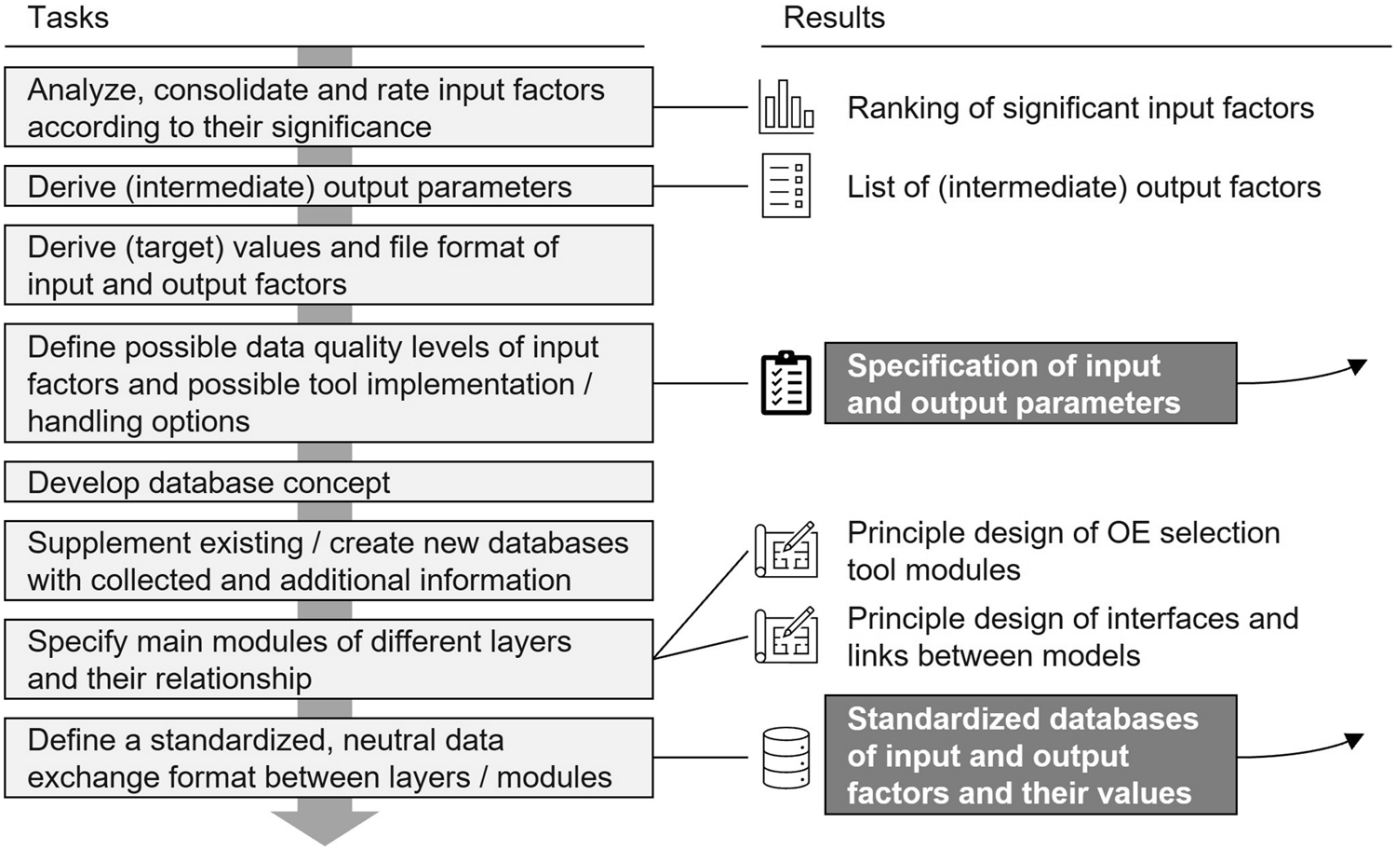
Tasks and results of stage *Conception of the basic evaluation systematic*

The main result of the second stage *Conceptualize* is a specification of input and output parameters and a definition of how they are stored and accessed (see Fig. 5). This stage focuses on the specification of the knowledge storage and sets the basis for the design of the other layers.

First, the input factors gathered in the previous stage are analyzed, consolidated, and evaluated according to their perceived importance for the simulation outcomes, culminating in a ranking of these factors that serves as a guideline for the further decision-making process. Higher-ranked input factors should be prioritized and implemented, while lower-ranked factors may be neglected. Output parameters are then derived and compiled in a structured list. Intermediate output parameters will exist in between the layers to serve as input factors for the subsequent layer. Furthermore, target values are set for the input and output factors, and the file format is defined to ensure compatibility and consistency between the simulation modules of the selection tool.

**Fig. 6:**
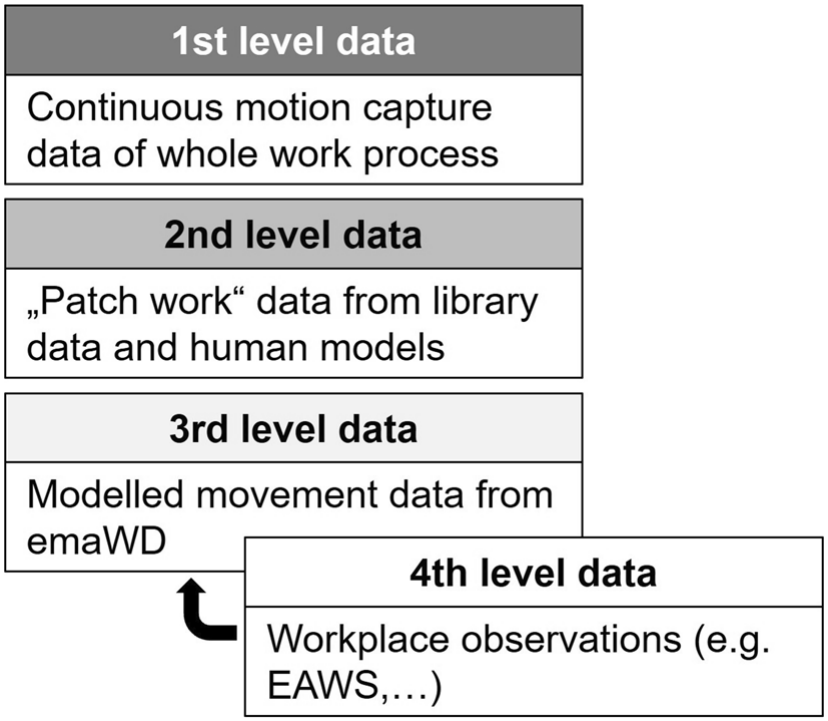
Different data level for movement data

The data quality differs based on how it is collected, prepared, and what information it contains. Exemplary, Fig. 6 shows the different level for movement data. Wherever applicable, a level description must be developed for all relevant other input factors to improve the model’s applicability. On the one hand, the input and output characteristics will be determined based on existing study data or parameters or by querying internet databases. On the other hand, the guidelines for describing industrial tasks offer different data characteristics. Hence, it should be possible to integrate input data on different levels into the simulation to allow a broad-field application of the selection tool after development. By ensuring this, companies will get guidance from available data.

For example, regarding the input factor of task and workplace properties, first-level data is the most accurate and consists of a continuous motion capture of the whole task or work process. In that case, joint kinematics and three-dimensional positional data will be used as input factors for the process and biomechanical simulation. It also integrates specific user characteristics, relevant process variables and loads. However, integrating motion capture systems in running industrial work processes is time-consuming and might stop production for a certain time. Thus, this kind of data will not be available for all workplaces. Fourth-level data contains the least information about user and task characteristics. This data is gathered by work assessment using "pen and paper"-methods such as the *EAWS*. It will be transformed into a movement model in a suitable way that is useful for a user-specific process and biomechanical simulation. For that, an exoskeleton-specific classification of tasks must be developed to comprehend the various support scenarios and movements within it. Theoretical approaches for classification tasks and exoskeletons will form the basis for that [42]. Second- and third-level data represent corresponding intermediate steps of the data preparation. Wherever applicable, a level description must be developed for all relevant other input factors to improve the model’s applicability.

### Design: co-simulation model design

**Fig. 7:**
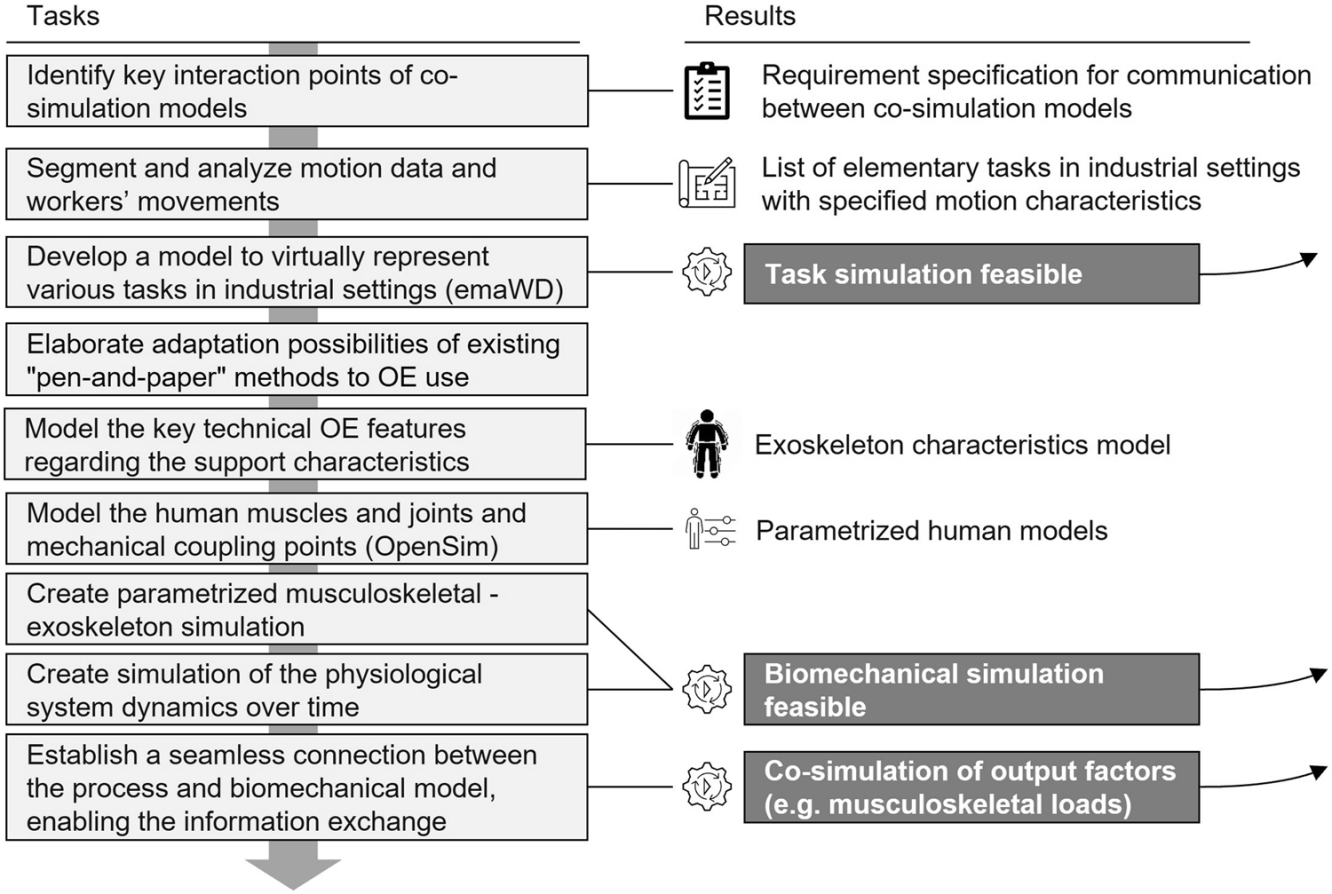
Tasks and results of stage *Co-Simulation model design*

The design phase consists of designing the simulation layer (see Fig. 7) and the decision layer. The simulation layer is developed before the decision model is created since the output parameters from the simulation are the key input parameters for the decision model. As stated above, a distinction is possible between the task simulation and the biomechanical simulation, which comprises a musculoskeletal-exoskeleton simulation and a physiological dynamics system simulation. A model of the OE characteristics that is used in the simulation completes the simulation layer.

Initially, while the previous stage focuses on the interaction in between the tool layers, this stage starts by identifying key interaction points between co-simulation models to establish requirements for communication between them, ensuring seamless integration and data exchange within this layer.

Subsequently, the focus shifts towards the detailed analysis of motion data, workers’ movements, and occurring loads within industrial settings. These movements are categorized and characterized through segmentation and analysis, yielding a comprehensive list of elementary tasks commonly encountered in industrial environments, extending and detailing the ones found in the literature (e.g., [19]). Each task is examined, with its unique movement characteristics documented and cataloged. This categorization serves as a key precursor to map the different data levels within the simulation environment [86]. The movements are then implemented in a virtual model to allow the task simulation, while the representation of both primary (e.g., lifting the arm, bending the back) and secondary activities (e.g., workplace transfers) by the model is essential. Furthermore, task-specific characteristics (e.g., loads with their motion trajectories, object sizes) will be added. Based on preliminary work, the software *emaWD* is a promising fit for simulating the activity and will be used [87].

Simultaneously, efforts are directed towards adaptating existing "pen and paper"-methods to the unique context of OE use, ensuring their seamless integration within the simulation environment. This will improve applicability and acceptance by users and ergonomists. Moreover, key technical properties of OE support characteristics (e.g., foce path, level of support, and torque curves) are modeled and synthesized, culminating in developing a comprehensive OE characteristics model. It should be suitable for mapping the functionalities of passive OEs (fixed torque curves) and active OEs (e.g., movement- and task-dependent torque curves). Therefore, at the mechanical coupling points to the human model, user movements and postures must be modeled as a function of movement tendencies. The OE model serves as a knowledge storage of essential characteristics, encapsulating the diverse functionalities and capabilities of OEs across various industrial applications. By doing so, the presented methodology goes beyond existing co-simulation approaches from related work (e.g., [37]) by considering extensive databases as input for the simulation as well as providing a decision model following the co-simulation, thus promoting the targeted selection of OEs.

**Fig. 8:**
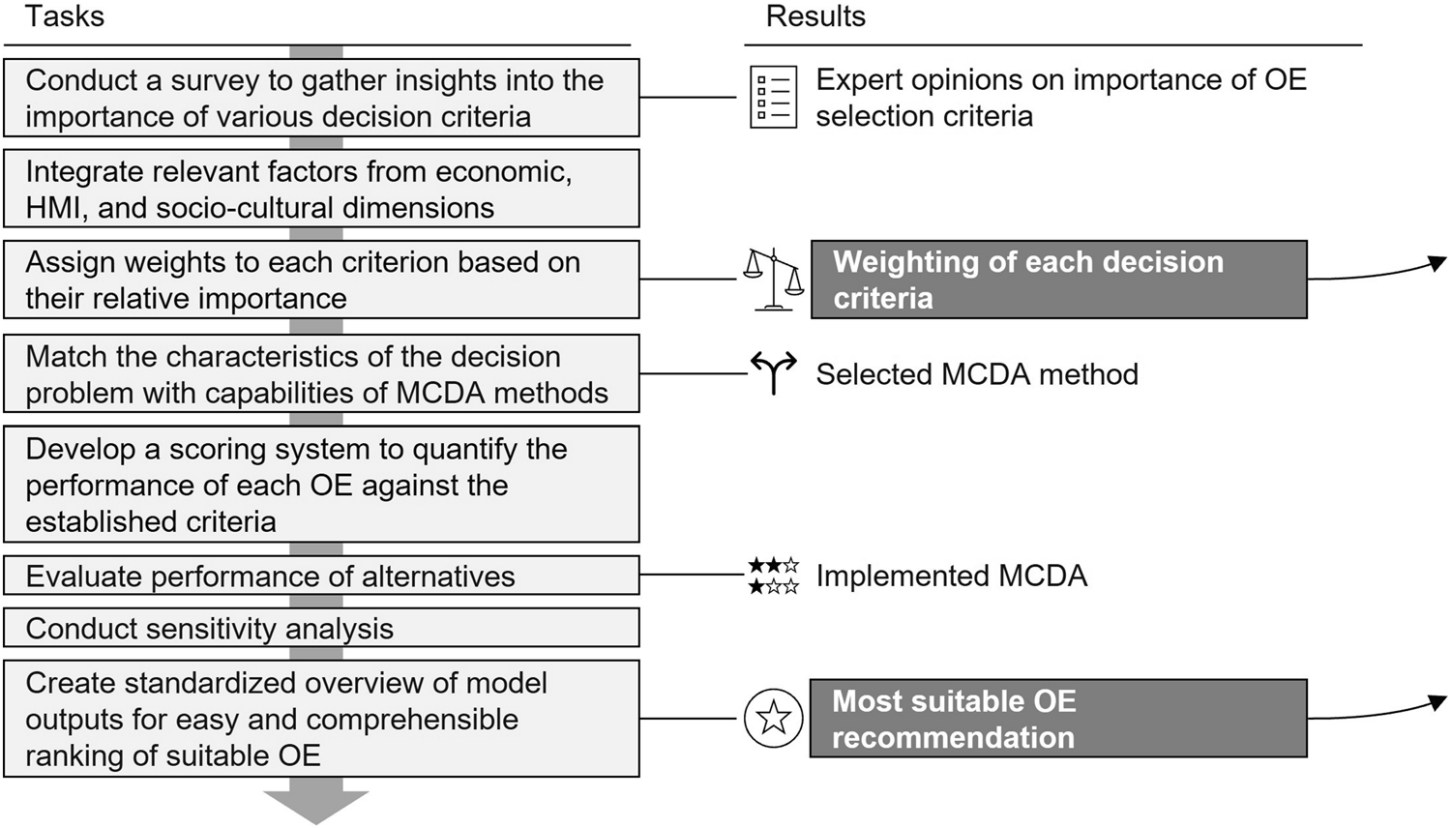
Tasks and results of stage *Decision layer design*

Next, human muscles, joints, and mechanical coupling points are modeled using *OpenSim* to create parametrized human models. Anthropometric scaling should be considered, including body measurements, masses, and inertia. Existing biomechanical models of human–exoskeleton interaction, as described in Sect. 2, serve as foundational references and integration points within the proposed co-simulation. The parameterized human model serves as the key element of the musculoskeletal-exoskeletal simulation. Utilizing the models offers insights into muscular activation, joint reaction forces, and potential musculoskeletal stress in specific applications. Furthermore, it enables the dynamic simulation of physiological system dynamics over time, which allows the simulation of time-relevant changes in the user’s ability to work, e.g., due to fatigue, and thus the calculation of dynamic output factors.

The quantification of fatigue is incorporated by integrating user performance capacity into the biomechanical model at different levels, similar to the approach used for movement data described in Section5.2. At the first level, performance data is obtained from direct physiological and biomechanical measurements, such as maximal oxygen uptake and isometric maximal force for various movements, providing highly accurate input for individualized simulations. The second level involves general fitness and strength estimations based on quantified data, which may include physical assessments or work-related performance metrics. At the third level, a qualitative estimation is applied, categorizing workers into broad fitness and strength levels (low, medium, high) using standard demographic factors such as age. The fatigue model itself is based on empirical measurement data from healthy young adults, with the flexibility to incorporate further refinements. This tiered approach ensures adaptability, balancing data availability and precision to provide realistic, scalable assessments of human performance and fatigue in industrial work environments.

The final step involves establishing a seamless connection between process and biomechanical models, facilitating information exchange, and enabling the co-simulation of output factors such as musculoskeletal loads.

### Design: decision layer design

Developing and implementing of a robust decision-making framework for selecting suitable OEs relies on various key steps, including defining criteria weighting, implementing a multi-criteria decision analysis, and creating a standardized overview of model outputs (see Fig. 8).

While defining weighting for criteria, various stakeholders (e.g., exoskeleton manufacturers, users, accident insurance organizations, employers’ liability insurance associations, and scientists) are surveyed to determine the importance of each criterion. This survey seeks to gather insights into the relative significance of criteria across the simulation output, economic, and soft factors. In that way, each criterion can be weighted. These weights reflect the decision-maker’s preferences and the importance of each criterion.

Among these criteria, qualitative aspects such as usability, comfort, and user acceptance play a crucial role in the decision-making process. To ensure consistency and comparability across different OEs, standardization of these aspects is essential. A structured evaluation process is employed to assess qualitative attributes, utilizing predefined scoring models based on expert evaluation, user feedback, and performance observations. Expert evaluation ensures objective assessments through predefined criteria established by specialists in ergonomics and biomechanics. User feedback surveys, such as System Usability Scale, help quantify subjective perceptions in a repeatable manner [88]. Additionally, observational and performance-based metrics, including donning/doffing time, freedom of movement, and postural stability, contribute to quantifying usability aspects that are otherwise difficult to measure.

However, certain trade-offs exist. Loss of individualization poses a challenge, as predefined scoring models may not fully account for occurring variations. Furthermore, weighting inconsistencies can arise, as different stakeholders may prioritize qualitative factors differently-for example, comfort may be more critical for long-duration tasks, whereas ease of movement may be more relevant for dynamic work environments. Nevertheless, by incorporating standardized qualitative assessments into the decision process, the methodology ensures structured and objective evaluation while acknowledging the importance of adaptability.

The next step involves the set-up of a decision model based on MCDA. Selecting an appropriate MCDA method ensures the reliability and effectiveness of the decision outcomes since each method is characterized by distinct principles, assumptions, advantages, and limitations. The capabilities of MCDA methods, e.g., *the Analytic Network Process (ANP)* [89], or *Preference Ranking Organization METHod for Enrichment Evaluation (PROMETHEE)* [90] are examined and matched to the characteristics of the decision problem. Factors such as the number of criteria and alternatives, the presence of uncertainty, the type of data available, and stakeholder preferences play a key role in determining the suitability of each method.

Subsequently, a scoring system can be developed to assess the performance of each OE against the established criteria, enabling a systematic comparison of alternatives. Furthermore, a sensitivity analysis must be conducted to evaluate the robustness of the decision model to variations in criteria weights or input data, ensuring the reliability and stability of the decision-making process.

**Fig. 9:**
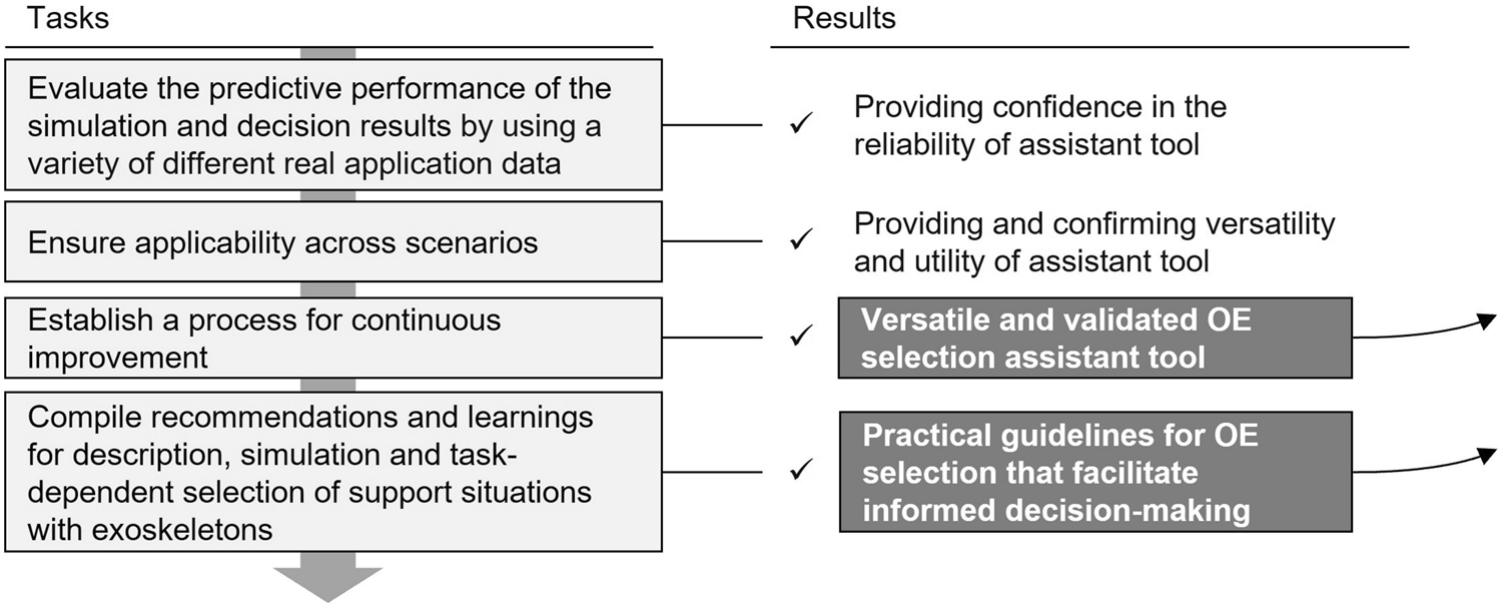
Tasks and results of stage *Decision layer design*

Finally, a standardized overview of model outputs is developed to facilitate the comprehensible ranking of suitable OEs. This involves synthesizing the results obtained from the MCDA into a clear and concise format that enables stakeholders to discern the relative merits of different OEs. By providing a standardized overview, stakeholders can make informed decisions based on the synthesized information. These activities culminate in the design of the ranking layer.

### Validation and optimization

The last stage is shown in Fig. 9. It ensures that the proposed solution meets the specified requirements and functions as intended. It involves testing and validating the predictive performance of the simulation and decision results generated by the tool using a diverse range of real-world application data, confirming its reliability, versatility, and utility. Since the development of OEs is still rapidly changing, new devices are designed, and the testing of existing devices is improved and enhanced, a continuous improvement process must be established to update the knowledge storage and databases. Hence, the selection tool remains adaptable and responsive to new technological developments and user feedback. This iterative approach allows continuous refinement and improvement of the tool’s functionality, increasing its effectiveness and user satisfaction.

Ultimately, the culmination of these efforts leads to creating a versatile and validated OE selection tool. Equipped with practical guidelines and recommendations derived from real-world data and simulations, the tool empowers users to make informed decisions regarding selecting and implementing OEs in their workplaces. By facilitating knowledge-based decision-making, the tool contributes to improving workplace ergonomics, safety, and productivity, ultimately benefiting both workers and employers alike.

## Implications for research and industry

**Table 2:** Summary of implications regarding research and industry

Implications regarding research	Implications regarding industry
Development of a comprehensive co-simulation-based approach for the evaluation and selection of OEs for industrial scenarios Creation of a knowledge-based simulation to merge human workers’ behavior and anthropometry in work activities with associated support technology Improved understanding, parametrization, and quantification of various input factors on OEs simulation, leading to more accurate and tailored recommendations for their use in specific industrial contexts Establishment of a systematic approach for comparing heterogeneous OEs Potential for developing novel simulation tools that can be applied beyond the scope of OE evaluation, benefiting other areas assistive technology	Enhancing compatibility of different OEs within workplace scenarios List of important tasks and steps for deciding on a specific OE Providing a simulation approach and improved evidence-based recommendations for informed OE selection Opportunity for industry stakeholders to contribute to refining research methodologies Contribution to awareness of the complex selection process and existing target conflicts in the industrial domain

The research outcomes present significant implications for both academic research and industrial practice in the domain of OEs. These are detailed below and summarized in Table 2.

Regarding research, the results promote the development of a comprehensive co-simulation-based methodology for the evaluation, and selection of OEs in industrial scenarios. This methodology integrates various input factors, including the human movements, the task and workplace properties and anthropometry of human workers, with the associated assistive technology, leading to a more sophisticated understanding of the performance of OEs in real-world contexts. By applying the methodology, these input factors are further parametrized and quantified. Importantly, the methodology will guide other researchers in developing simulation tools for new wearable assistive devices that benefit other areas of human–machine interaction and wearable assistive technology.

In the industrial domain, the research has the potential to improve the compatibility of different OEs in specific workplace scenarios. The simulation approach facilitates informed decision-making in the selection of OEs. Additionally, the study provides a comprehensive list of important tasks and steps for deciding on a specific OE, offering clarity and guidance in decision-making. Furthermore, companies receive an overview of relevant literature for OE selection and, thus, evidence-based recommendations for action to increase the acceptance of OE technology. Furthermore, this research helps to raise awareness of the complex selection process and the existing target conflicts and promotes the refinement of research methodologies, allowing industry stakeholders to actively contribute to the ongoing evolution of OE technology.

## Conclusion

In conclusion, this paper addresses the critical need for a systematic approach to select OEs in industrial settings, where the interaction between humans and technology is increasingly prevalent. The existing selection processes often rely on recommendations by sales representatives. A lack of comprehensive testing and evaluation leads to potential issues with the acceptance, task suitability, and effectiveness of the selected OE. Recognizing these challenges, this research proposes a simulation-based methodology for OE selection, leveraging knowledge-based techniques and co-simulation models.The primary objective of the methodology is to develop a tool to facilitate the selection of suitable OEs based on a comprehensive understanding of user anthropometrics, task characteristics, and OE designs. Regarding the first research question, existing approaches for selecting a specific OE are shown, and key criteria driving the selection process are derived. Various approaches and methods are presented that build a knowledge storage for developing a knowledge-based selection tool. The knowledge storage will combine ergonomic and biomechanical assessments of OEs, the description, evaluation, and structure of manual work activities and their characteristics as well as key findings on OE features. These results will be used when the proposed methodology is applied, as they form the dominant input factors for the knowledge-based selection.

Through the systematic delineation of stages and activities, this work fosters transparency, reproducibility, and rigor in developing the selection tool. Using a stage-gate process ensures thorough evaluation and validation at each stage, culminating in the successful implementation OE selection tool. The presented structure of the methodology with its five stages, tasks, and desired (intermediate) results represents the answer to the second research question. Central to the methodology is the development of an interdependent task and biomechanical simulation. This enables the modeling and evaluation of OE performance across diverse task scenarios and users. Additionally, the decision model incorporates a multi-criteria decision analysis to systematically rank suitable OE based on predefined criteria. By defining weighting for criteria, implementing evaluation methods, and creating standardized overviews of model outputs, the decision model will offer a structured framework for informed decision-making in OE selection. During the discussion of the results, implications for research and industry resulting from a knowledge-based OE selection are derived, which provides the answer to the third research question.

Looking ahead, while this research presents the overall framework for the selection process, future work will focus on detailing and carrying out each stage of the methodology. With each implemented stage, tangible benefits to companies arise. Risks associated with investing in inappropriate OE solutions and optimizing resource allocation through informed decision-making processes are minimized. This includes the development of a MCDA for OE selection, contributing to the refinement of industry-wide standards and guidelines. Ultimately, these efforts aim to foster increased adoption and acceptance of OE technology in industrial settings.

An essential next step is to demonstrate the decision process in a real-world application to further assess the methodology’s applicability, effectiveness, and validity. While the individual components of the framework - such as biomechanical simulation, process modeling, and MCDA - have already been developed and validated separately, their combined implementation within a fully integrated co-simulation environment remains untested. Since these subsystems function with a defined level of accuracy, their interactions, dependencies, and overall predictive capabilities must be systematically analyzed. This integration will provide insights into which data levels yield meaningful results for different aspects of the simulation, allowing for a balanced trade-off between data availability, model precision, and decision-making reliability. Future work will focus on verifying whether the combination of these elements leads to a coherent and interpretable decision-making process or introduces additional complexity that must be addressed.

Despite its contributions, this research is not without limitations. Firstly, the scope of the study may be constrained by the availability and quality of existing data in the literature. The current lack of standardized evaluation criteria and methodologies across different approaches might hinder the comparability of findings. Furthermore, the absence of comprehensive research investigating the effectiveness and usability of OEs in real workplace settings poses a challenge, as laboratory-based studies may have limited relevance to industrial contexts. The dynamic nature of OE development may also render some aspects of the research less applicable over time. These limitations underscore the need for continued refinement and validation of the methodology to address evolving challenges in the field of OE research and application.

In summary, the proposed methodology offers a systematic and comprehensive approach to address the complex challenges associated with OE selection in industrial settings. By integrating simulation-based techniques, knowledge storages, and decision-making frameworks, it will become possible to empower industry practitioners with the tools and insights needed to enhance safety, productivity, and well-being in the workplace by using OEs.

## Data Availability

No datasets were generated or analysed during the current study.
